# People with higher relationship satisfaction use more humor, valuing, and receptive listening to regulate their partners’ emotions

**DOI:** 10.1007/s12144-023-04432-4

**Published:** 2023-03-10

**Authors:** Sarah A. Walker, Rebecca T. Pinkus, Sally Olderbak, Carolyn MacCann

**Affiliations:** 1 School of Psychology, University of Sydney, Sydney, NSW Australia; 2 Institute of Psychology and Education, Ulm University, Ulm, Germany

**Keywords:** Emotion regulation, Relationship satisfaction, Humor, Valuing

## Abstract

The emotional experiences you have with a romantic partner shape how satisfied you are in your relationship. Engaging in attempts to make a romantic partner feel better is linked with better relationship outcomes. However, it is not yet clear which specific processes people use to regulate their partners’ emotions, nor which processes are most strongly linked with relationship satisfaction. In the current study of 277 individuals (55% female), we tested the extent to which eight extrinsic emotion regulation processes (expressive suppression, downward social comparison, humor, distraction, direct action, reappraisal, receptive listening, and valuing) predict relationship satisfaction. Six of the eight processes showed significant positive correlations with relationship satisfaction, with the strongest associations for valuing (*r* = .43), humor (*r* = .33), and receptive listening (*r* = .27). Relative weights were significant only for valuing, humor, and receptive listening, suggesting that these are the most important predictors of relationship satisfaction. Results are discussed in terms of the distinction between intrinsic and extrinsic regulation processes and the potential importance of motives for regulation.

**Supplementary Information:**

The online version contains supplementary material available at 10.1007/s12144-023-04432-4.

Emotion regulation refers to the processes people use to influence the intensity, duration, and expression of emotions (Gross, 2002). While emotion regulation is typically studied as an intrinsic process (how someone regulates their own emotions; Gross, 1998), there is substantial evidence that people also regulate the emotions of others (Niven, Macdonald et al., 2012; Nozaki & Mikolajczak, 2020; Williams et al., 2018). For example, you may try to make your romantic partner feel better if you noticed they have had a stressful day. This attempt to modulate the emotional experience of another person is understood as ‘extrinsic emotion regulation’ (Nozaki & Mikolajczak). Recent research shows that attempting to make your partner feel better (regulating their emotions) is linked to higher relationship satisfaction (e.g., Kinkead et al., 2021). However, it is not yet clear whether some extrinsic emotion regulation strategies may be more beneficial than others. For example, using positive reappraisal (encouraging your partner to re-interpret events more positively) may be a more effective approach than using expressive suppression (encouraging your partner to hide their emotions). As yet, there is no research comparing multiple extrinsic regulation processes in romantic relationships. We address this gap by examining the extent to which eight discrete extrinsic emotion regulation processes predict romantic relationship satisfaction.

## Extrinsic emotion regulation

Emotion regulation is a core feature of our emotional lives and is important for personal wellbeing (Gross & John, 2003), workplace wellbeing (Fan & Wang, 2022; Greenier et al., 2021) and relationship quality (Niven et al., 2015; Williams et al., 2018). Research distinguishes between intrinsic (regulating one’s own emotions), extrinsic (regulating the emotions of another person), and interpersonal emotion regulation (regulating one’s own emotions through interpersonal social interactions) (Nozaki & Mikolajczak, 2020; Zaki & Williams, 2013). Both intrinsic and extrinsic emotion regulation can be represented by the Extended Process Model of Emotion Regulation (Gross, 2015) which is a theoretical framework used to understand how people regulate their own emotions, and the emotions of others. The model consists of three stages: (a) Identification. An individual must first recognize the need for regulation by inferring the emotional state of the target person, assigning a value to the emotional state, and then forming a regulation goal, (b) Selection. An individual perceives, evaluates, and chooses the most effective regulation processes, and (c) Implementation. An individual operationalizes the selected processes and evaluates the feasibility and outcomes of the specific tactic/s chosen.

There is a large extant literature exploring the range of processes people use to influence their own emotional experiences (Webb et al., 2012) with some strategies found to be more effective than others. For example, cognitive reappraisal (reframing a situation in a more positive light) has been found to be generally more effective than expressive suppression (not physically showing one’s emotions). However, less is understood about the processes people use to influence the emotional trajectory of others – known as *extrinsic emotion regulation*. Extrinsic emotion regulation is typically measured in terms of two broad goals: *affect improving* (i.e., enhancing someone else’s emotions) and *affect worsening* (i.e., diminishing someone else’s emotions; Niven et al., 2011). The primary conceptual model of extrinsic regulation describes multiple different processes that could be used to improve or worsen affect (Niven et al., 2009). For example, I could use *humor* to make a friend laugh, or use *valuing* to make an employee feel proud by telling them how important and valued their work is. Importantly, according to Nozaki and Mikolajczac (Nozaki & Mikolajczak, 2020), extrinsic emotion regulation has three core features: (1) the regulator must have the goal of influencing the emotional trajectory of another person, (2) the extrinsic regulation goal can either increase or decrease negative or positive emotions, and (3) extrinsic regulation is distinguished from other related constructs such as empathy and emotional contagion.

The expression of negative emotions can influence an emotional and behavioural responses in others (Keltner & Haidt, 1999; Keltner & Kring, 1998) which may motivate a sympathetic reaction toward the person expressing the negative emotion (Lench et al., 2016). Attempting to regulate the emotions of another person can result in the development of stronger relationships, and deeper trust in those relationships (Keltner & Haidt, 2001; Keltner & Kring, 1998; Niven et al., 2012). Evidence suggests that by regulating the emotions of others, an individual may experience an increase in their own positive affect, thereby benefiting from the act of helping others regulate their emotional experience (Austin et al., 2018; Austin & Vahle, 2016; Niven et al., 2012).

Although there is burgeoning interest in examining the broad categories of affect improving, and affect worsening, little is yet understood about the relative benefits of different, discrete regulation processes that people use to modulate the emotional experiences of others (MacCann et al., 2019) and the impact on regulation on the regulators perception of relationship satisfaction. To address this gap, the current study examines the eight regulation strategies of the Regulation of Others’ Emotions Scales (ROES; MacCann et al., 2019). Each process can be classified in terms of the degree of engagement with the target person (low, moderate, or high) and the focus of process (disengagement-focused, diversion-focused, change-focused, and relationship-focused; Table 1). Intrinsic emotion regulation research clearly shows that some processes are more effective than others (Mazzuca et al., 2019). We will explore if extrinsic regulation processes similarly have different effects on a key outcome among romantic partners - relationship satisfaction.

**Table 1:** Description of extrinsic emotion regulation processes included in the Regulation of Others’ Emotions Scales (ROES; MacCann et al., 2019)

Process	Description	Focus	Emotional Engagement
Expressive suppression	The regulator encourages the target to avoid verbally or physically expressing their emotions.	Disengagement	Low
Downward comparison	The regulator compares the target’s situation to someone in a worse situation.	Disengagement	Low
Humor	The regulator tries to increase positive affect by making the target laugh (e.g., telling a joke or sharing a funny story).	Diversion	Moderate
Distraction	The regulator attempts to reduce negative affect by refocusing the target’s attention away from the emotional event.	Diversion	Moderate
Direct action	The regulator directly changes the target’s situation to reduce negative affect	Change	Moderate
Positive reappraisal	This occurs when the regulator encourages the target to shift the way they think about a situation in order to increase positive affect.	Change	High
Receptive listening	The regulator encourages the target to express their emotions to help reduce negative affect.	Relationship	High
Valuing	The regulator expresses how much the target is valued and special to increase positive affect.	Relationship	High

*Note*: ER = extrinsic emotion regulation

## Relationship satisfaction

Romantic relationships represent a core feature of people’s lives. Characteristics such as commitment, love, trust, communication, security, and emotional support help people evaluate how satisfied they feel in their relationship (Fletcher et al., 2015; Hendrick et al., 1998). Romantic relationships inevitably involve the ebb and flow of negative and positive events that influence both partners’ wellbeing (Antonucci et al., 2001). For example, when a person’s needs and expectations are not met in the relationship, this can lead to increased negative affect and adverse mental and physical health outcomes for both partners (Bravo et al., 2017; McNulty et al., 2021; Whisman, 2007). Furthermore, the longer adverse outcomes persist, the more likely relationship satisfaction will decline, potentially leading to a breakdown of the relationship (Bravo, 2017; McNulty et al.,^2021^). In contrast, when a person’s needs and expectations are met in the relationship, and arising conflict is met with effective communication and emotional support, this can lead to increased positive affect, wellbeing, self-esteem, life satisfaction, and relationship satisfaction (Antonucci et al., 2001; Pateraki & Roussi, 2013; Voss et al., 1999). Therefore, it is essential to examine and understand the factors that improve and maintain relationship satisfaction as well as identify factors that undermine relationship satisfaction.

## Extrinsic emotion regulation and relationship satisfaction

Most research on emotion regulation in romantic relationships examines how people regulate their own emotions (rather than their partner’s emotions). However, one partner’s efforts to regulate their own emotions (intrinsic regulation) can also positively or negatively impact their partner’s emotions (extrinsic regulation; Proulx et al., 2007). We know that intrinsic emotion regulation affects romantic relationship functioning and satisfaction (Ben-Naim et al., 2013; Gross & John, 2003; Vater & Schroder-Abe, 2015). We also know that some processes are better than others. For example, intrinsic expressive suppression (suppressing one’s own emotions) leads to lower relationship satisfaction over time (Gross & John, 2003; Impett et al., 2012) whereas intrinsic cognitive reappraisal processes relate to higher relationship quality (Ruso et al., 2019). Similarly, downward comparison in romantic relationships tends to result in lower positive feelings (Pinkus et al., 2008). It is feasible that *extrinsic* expressive suppression, reappraisal, and downward social comparison might have similar effects on relationship satisfaction.

The two relationship-focused processes (valuing and receptive listening) centre on the relationship between the regulator and the target and involve high levels of engagement with the other person. Intuitively, relationship-focused processes should relate to people’s satisfaction in their relationships. There is evidence that empathic listening improves the other person’s emotional response to negative events in terms of both subjective feelings and physiological response (Seehausen et al., 2012). Moreover, using receptive listening relates to increased closeness and reduced loneliness (Nils & Rimé, 2012; Pauw et al. 2018).

There is also evidence that humor may relate to relationship satisfaction. Humor tends to improve positive emotions (Fredrickson & Levenson, 1998) and distract from negative emotions (Strick et al., 2009). In fact, although humor involves less engagement with the target, using humor is positively associated with relationship quality (Cann et al., 2009; Carstensen et al., 1995; Kurtz & Algoe, 2015). Although both direct and indirect support have been shown to positively relate to relationship satisfaction, direct action tends to be less effective than indirect action and may also increase negative affect (Girme et al., 2018).

Given the findings above, we hypothesize that humor, direct action, distraction, reappraisal, receptive listening, and valuing will relate to higher relationship satisfaction, whereas downward comparison, and expressive suppression will relate to lower satisfaction. Because the eight extrinsic regulation strategies are highly inter-correlated, we will examine relative weights analysis (Tonidandel et al., 2009) as well as multiple regression in evaluating the relative importance of each of the eight processes.

## Method

### Participants

Participants (*N* = 277, 55% female, mean age = 36.8 years, *SD* = 12.21 years) were recruited through Prolific in May 2020, as part of a larger project examining wellbeing during the coronavirus pandemic. Most participants (*n* = 267, 96%) were in a romantic relationship with an opposite-sex partner. An additional 57 participants completed the protocol but were excluded based on pre-registered exclusion criteria (i.e., taking less than a third of the median response time, failing a data check item, stopping halfway through, showing invariant responding across 2 or more screens, or answering that they spoke English “not well” or “not at all; see https://aspredicted.org/blind.php?x=HSL_CMW).

### Materials

#### Regulation of others’ emotions scale^1^ (ROES; MacCann et al., 2019)

Partner frame-of-reference instructions were used, with reference to the coronavirus pandemic, which put stress on relationships (i.e., “Since the coronavirus outbreak, I do the following things to MAKE MY ROMANTIC PARTNER FEEL BETTER”). Eight subscales were assessed with four items each (expressive suppression, downward social comparison, distraction, humor, direct action, reappraisal, receptive listening, and valuing). Sample items include “I ask them to put a brave face on” (expressive suppression) or “I do what I can to find an answer for them” (direct action). Participants rated each item on a 6-point scale, from 1 (*Strongly Disagree*) to 6 (*Strongly Agree).*

#### Relationship satisfaction (Hendrick, 1988)

Instructions requested participants rate each of the five items on a 7-point Likert scale, from 1 (*Strongly Disagree)* to 7 (*Strongly Agree*). Items included “How well does you partner meet your needs?” and “How often do you wish you hadn’t gotten into this relationship?” (reverse scored).

### Procedure

Participants were recruited from Prolific by seeing a study ad and provided written consent after viewing a participant information statement. All participants completed demographic questions and then several rating scales including the ROES and the relationship satisfaction scale (see preregistration for full list of measures not included in the current study). This study was approved by the Human Ethics Committee at the first author’s institution.

### Analysis

Three measures of the association of the eight regulation processes with relationship satisfaction were conducted: (1) Pearson’s correlations (*r*); (2) multiple regressions where the 8 processes predicted relationship satisfaction, and (3) relative weights analysis (RWA) to test the relative importance of each of the eight predictors. An RWA partitions the *R*^*2*^ into pseudo-orthogonal parts with each part representative of the relative contribution of each predictor variable (Tonidandel et al., 2009). While regression analyses can help identify the relationship between variables, relatively weights analysis provides a more detailed understanding of the relative importance of each variable in the model – particularly with multiple predictor variables, as it helps to identify which variables are most important in predicting the outcome. We interpret effect size of correlations and regression coefficients with respect to Cohen’s *r*, with.10, 0.30, and 0.50 considered as “small”, “medium” and “large” respectively (Cohen, 1988). Statistics were calculated using R 4.1.0 (R Core Team, 2021), effsize (v0.8.1; Torchiano, 2020), and RWA (v0.0.3; Chan, 2021). The full reproducible code is available in supplementary materials.

## Results

### Internal consistency and descriptive statistics

Table 2 presents the internal consistency indices, and descriptive statistics for the ROES subscales and relationship satisfaction. Mean scores were lowest for the disengagement-focused processes and highest for the relationship-focused processes. Internal consistency reliability for the ROES subscale scores were good. Confirmatory factor analyses were conducted to test model fit for the Regulation of Others’ Scale, and relationship satisfaction. There was good model fit for the eight-factor structure of the Regulation of Others’ Scale (χ^2^ = 714.50, *df* = 436, CFI = 0.994, SRMR = 0.060, RMSEA = 0.048, 90% CI = 0.042 − 0.054), and good fit for Relationship Satisfaction (χ^2^ = 27.08, *df* = 14, CFI = 0.999, SRMR = 0.03, RMSEA = 0.058, 90% CI = 0.23 − 0.91).

### Correlations

Relationship satisfaction was not significantly correlated with the two disengagement-focused strategies but showed significant positive correlations for the other six regulation strategies. The effect size was moderate-to-large for valuing, moderate for receptive listening and humor, and small-to-moderate for the remaining processes (distraction, direct action and reappraisal).

### Hypothesis testing

Regression coefficients were significant for valuing and humor only, with a moderate-to-large effect for valuing and a small-to-moderate effect for humor. The regression explained 23% of the variance in relationship satisfaction. Relative weights analysis (RWA) was used to test hypotheses. Relative weights were significant for valuing (which explained 48.5% of the *R*^*2*^), humor (21.2% of *R*^*2*^) and receptive listening (9.73% of *R*^*2*^), as hypothesized. However, the hypothesized relationships for downward comparison, expressive suppression, direct action, and reappraisal were not supported.

**Table 2:** Descriptive statistics, inter-correlations of regulation processes, and prediction of relationship satisfaction (correlations, multiple regression, and relative weights analysis)

	Descriptive Statistics			Inter-Correlations of Extrinsic Regulation Processes								Prediction of Relationship Satisfaction			
	*M*	*SD*	α	1	2	3	4	5	6	7	8	*r*	*β*	RW	%*R2*
1. Expressive Suppression	2.21	0.92	0.80									− 0.05	− 0.09	0.01	2.23
2. Downward Comparison	3.39	1.31	0.91	.61^***^								.06	0.03	0.00	0.81
3. Humor	4.83	0.88	0.89	.02	.15^*^							.33^***^	0.18^**^	0.05^*^	21.20
4. Distraction	4.45	0.86	0.83	.27^***^	.37^***^	.57^***^						.23^***^	− 0.05	0.01	5.56
5. Direct Action	4.11	0.94	0.77	.33^***^	.35^***^	.30^***^	.39^***^					.22^***^	0.10	0.02	8.76
6. Reappraisal	4.31	0.85	0.86	.32^***^	.52^***^	.28^***^	0.51^***^	.56^***^				.18^**^	0.03	0.01	3.19
7. Receptive Listening	5.07	0.77	0.83	− .18^**^	.06	.37^***^	.40^***^	.23^***^	.43^***^			.27^***^	− 0.10	0.02^*^	9.73
8. Valuing	5.03	0.81	0.89	− .09	.07	.44^***^	.44^***^	.27^***^	.29^***^	.67^***^		.43^***^	0.40^***^	0.11^*^	48.50
9. Relationship Satisfaction	3.54	0.38	0.92	− .05	.06	.33^***^	.23^***^	.22^***^	.18^**^	.27^***^	.43^***^				

^*^p < .05, ^**^p < .01, ^***^p < .001

## Discussion

Our results supported hypotheses based on extrinsic regulation research and theory (i.e., valuing, humor, and receptive listening significantly predicted greater relationship satisfaction) but not for the hypotheses based on prior research on intrinsic emotion regulation (i.e., downward comparison, expressive suppression, direct action, and reappraisal did not significantly predict relationship satisfaction). These results highlight the distinction between intrinsic and extrinsic emotion regulation - just because a process works for you does not mean it works for your partner.

### Valuing

Consistent with prior research, valuing was the strongest predictor of greater regulator relationship satisfaction. Complimenting and valuing one’s partner benefits both the giver and receiver of the compliments (Doohan & Manusov, 2004). What is less clear from our results is the direction of causation. Relationship satisfaction may facilitate the act of valuing one’s partner (where those who are more satisfied are more likely to use valuing to regulate their partner’s emotions). Alternatively valuing one’s partner may create positive feelings about the partner and relationship thus strengthening relationship satisfaction (Murray & Homes, 2009; Murray et al., 2010).

### Humor

Results for humor are consistent with prior meta-analytic findings where humor predicted greater relationship satisfaction (Hall, 2017). In fact, shared laughter within a relationship predicts not only relationship satisfaction, but also evaluations of closeness, relationship quality, and social support (Kurtz & Algoe, 2015). This is in line with research on humour and attraction which demonstrates that men are more likely to be attracted to women who value their wit, and in turn, women are attracted to someone who makes them laugh (Bressler et al., 2006; Hone et al., 2015).

### Receptive listening

Consistent with prior research, our results indicate that receptive listening positively predicts relationship satisfaction (Pasupathi et al., 1999; Seehausen et al., 2012). Disclosing negative emotions to one’s romantic partner may indicate trust and a desire to connect with the listener (Graham et al., 2008). This may increase feelings of closeness and trust between the listener and their partner (Graham et al., 2008; Kashdan et al., 2007; Von Culin et al., 2018), which ultimately enhances the listener’s perception of their relationship satisfaction.

In contrast, expressive suppression, downward comparison, distraction, direct action, and reappraisal did not significantly predict relationship satisfaction. The null results for expressive suppression deviate from prior research where expressive suppression was associated with lower relationship satisfaction (English et al., 2012; Sasaki et al., 2021; Vater & Schröder-Abé, 2015). However, this past research has examined *intrinsic regulation*. Suppressing one’s own emotions is qualitatively different to the verbally-mediated act of telling someone else to change their expression of emotion. Similarly, the prior research showing that cognitive reappraisal processes related to greater relationship satisfaction focused on *intrinsic regulation*. These results may not generalize to extrinsic processes. In fact, Niven et al. (2015) suggest that extrinsic reappraisal may be viewed negatively by targets—as ‘splaining’ their own thoughts and feelings to them.

The motivation underlying the use of disengagement-focused processes, such as expressive suppression, may influence the extent to which they predict relationship satisfaction (Niven et al., 2019). That is, when the regulator’s prosocial underlying motivation is to positively influence their partner’s emotions, then the usually negative impact of using a less emotionally-engaging process may be diminished (Niven et al., 2019). Conversely, when the motivation of the regulator in using disengagement-focused processes is egoistic, we may see disengagement-focused processes leading to lower relationship satisfaction in both the target and regulator.

## Limitations and future research

The results of this study may represent the perspective of partner-as-regulators who are already happy in their relationship. Because the regulators are satisfied with their relationship, they are willing to engage emotionally with their partner. On the other hand, the regulator’s belief in their ability to make their partner laugh, and/or value their partner, may increase the regulator’s confidence that they can regulate their partner’s emotions thus leading to greater feelings of relationship satisfaction (Kirby & Baucom, 2017). Similarly, concerning the use of disengagement-focused processes, the already strong relationship satisfaction of the partner-as-regulator may act as a buffer such that the use of disengagement-focused processes do not significantly relate to relationship satisfaction. As the results from this study are potentially bi-directional, it is not possible to derive causal explanations from this cross-sectional study. Future longitudinal research is needed to determine whether the use of emotionally engaging extrinsic regulation processes increase relationship satisfaction, whether existing relationship satisfaction encourages greater emotional engagement when regulating one’s romantic partner, or whether there is a feedback loop, consistent with Gross’ extended process model (Gross, 2015).

Additionally, future research may consider taking a dyadic approach to examine how much the discrete extrinsic emotion regulation processes used by the regulator predict relationship satisfaction of both regulator and target. Moreover, exploring the extent to which the regulator’s underlying motivation for regulation impacts not only the use of discrete extrinsic regulation processes, but also the extent to which the discrete processes and motivation interact to predict relationship satisfaction in both target and regulator is needed.

## Conclusion

This research is among the first to evaluate the extent to which eight discrete emotion regulation processes relate to the relationship satisfaction of the regulator. The results of this study demonstrates that using receptive listening, humor and valuing to make one’s partner feel better is associated with the regulator feeling more satisfied in their relationship.

## Electronic supplementary material

Below is the link to the electronic supplementary material.

Supplementary Material 1

Supplementary Material 2

## Footnotes

1 While the preregistration specified 10 extrinsic regulation processes, the latest version of the ROES instrument includes only 8 processes. We therefore report these 8 processes (rather than the 10 that were pre-registered).
